# Supplementary material for: Iguratimod Ameliorates the Severity of Secondary Progressive Multiple Sclerosis in Model Mice by Directly Inhibiting IL-6 Production and Th17 Cell Migration via Mitigation of Glial Inflammation
Source: Biology (Basel). 2023 Sep 7;12(9):1217. doi: 10.3390/biology12091217 (PMC10525689; doi:10.3390/biology12091217)
Supplement: Supplementary file 1 [file biology-12-01217-s001.zip › biology-2475252-supplementary.pdf]

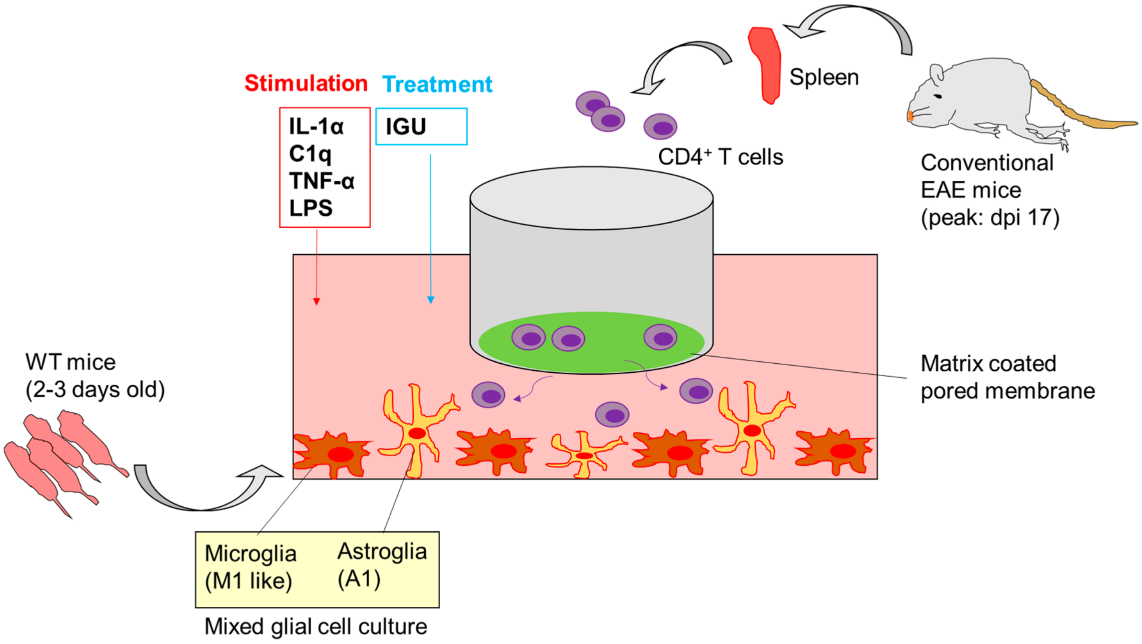

Figure S1. Schematic model of the *in vitro* migration assay. Mixed glial cell culture was prepared from newborn wild-type mice. The mixed glial cells were seeded in the lower transwell chambers with or without stimulation (IL-1α, C1q, TNF-α, LPS) and IGU. Magnetically separated CD4$^+$ T cells from acute EAE mice were loaded in the upper chamber. After 24 h of incubation, CD4$^+$ T cells that migrated to the lower chamber were counted.

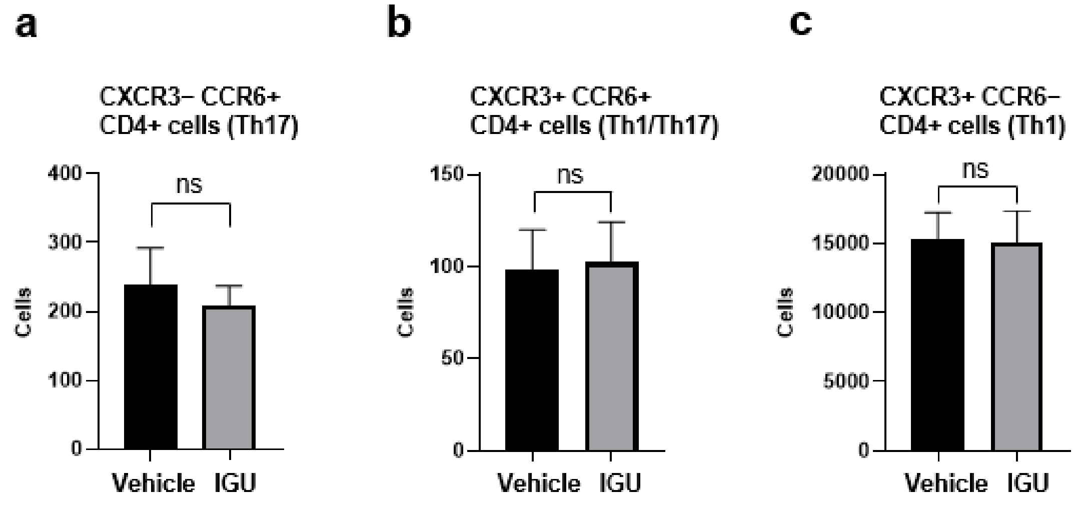

Figure S2. Magnetically separated CD4^{+} T cells were incubated with IGU or vehicle for 24 h (*n* = 3 per group). Statistical analyses were performed using an unpaired *t*-test. No significant differences were found between the groups.

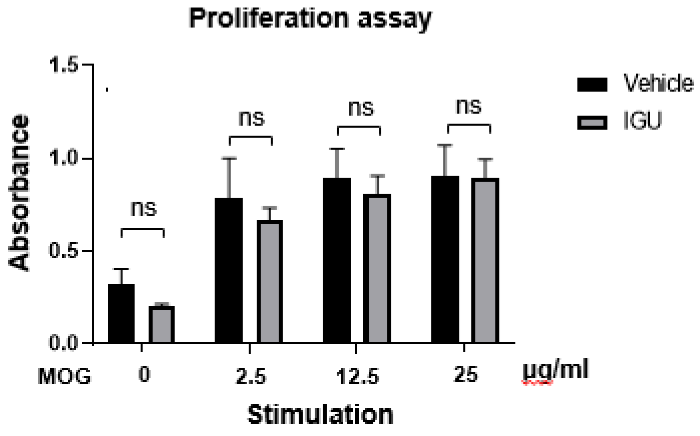

Figure S3. Splenic T cell proliferation assayed by bromodeoxyuridine (BrdU) incorporation at different myelin oligodendrocyte glycoprotein peptide concentrations (0, 2.5, 12.5, 25 μg/ml) in IGU-treated and vehicle-treated mice (*n* = 3 per group). Statistical analyses were performed by one-way ANOVA. No significant differences

were found between the groups.

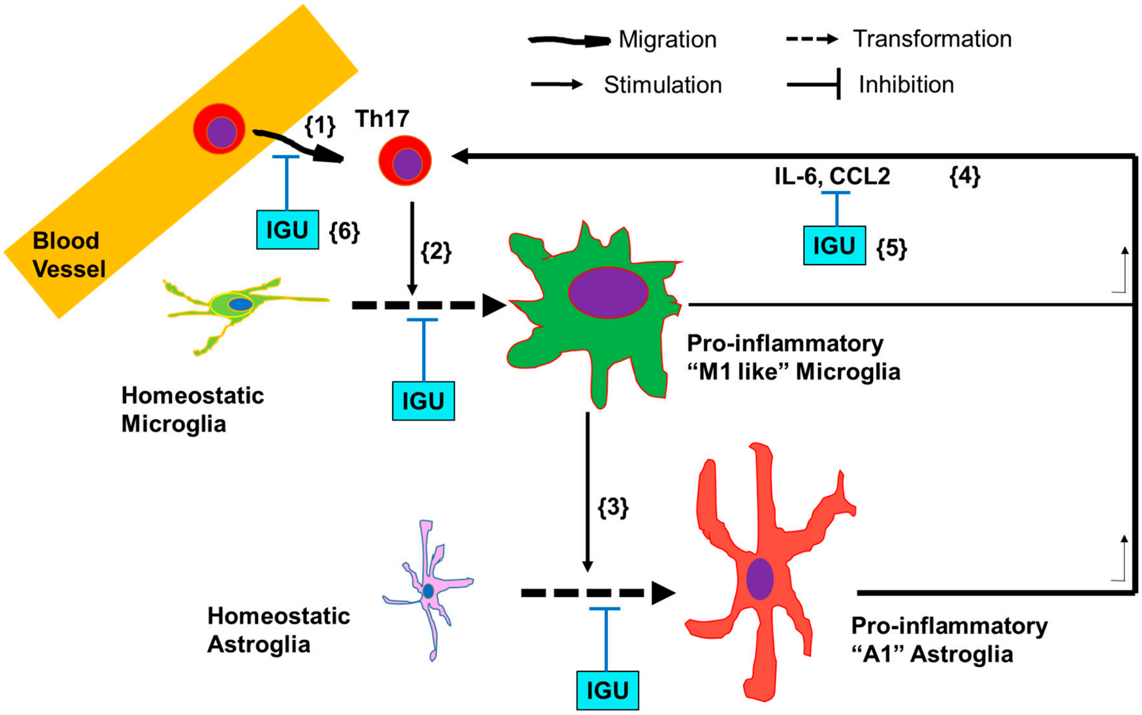

Figure S4. Schema for the mechanistic function of IGU in the amelioration of glial inflammation in pEAE lesions. At the beginning of multiple sclerosis, Th17 cells infiltrate through the blood-brain barrier from blood vessels {1}. Th17 cells induce a reactive M1-like phenotype in microglia {2}. Reactive M1-like microglia induce an A1 phenotype in astroglia {3}. Cytokines/chemokines, such as IL-6 and CCL2, secreted from glial cells induce Th17 cell migration {4}. Oligodendroglia-specific Cx47-inducible conditional knockout (*Cx47* icKO) increased A1 astroglia and M1-like microglia during both the acute and chronic phases. Moreover, Th17 cell migration was increased by *Cx47* icKO. IGU inhibited activated glial inflammation and cytokine/chemokine release, in particular IL-6 and CCL2 release {5}, which decreased

Th17 cell migration into the CNS {6}. IGU: iguratimod.

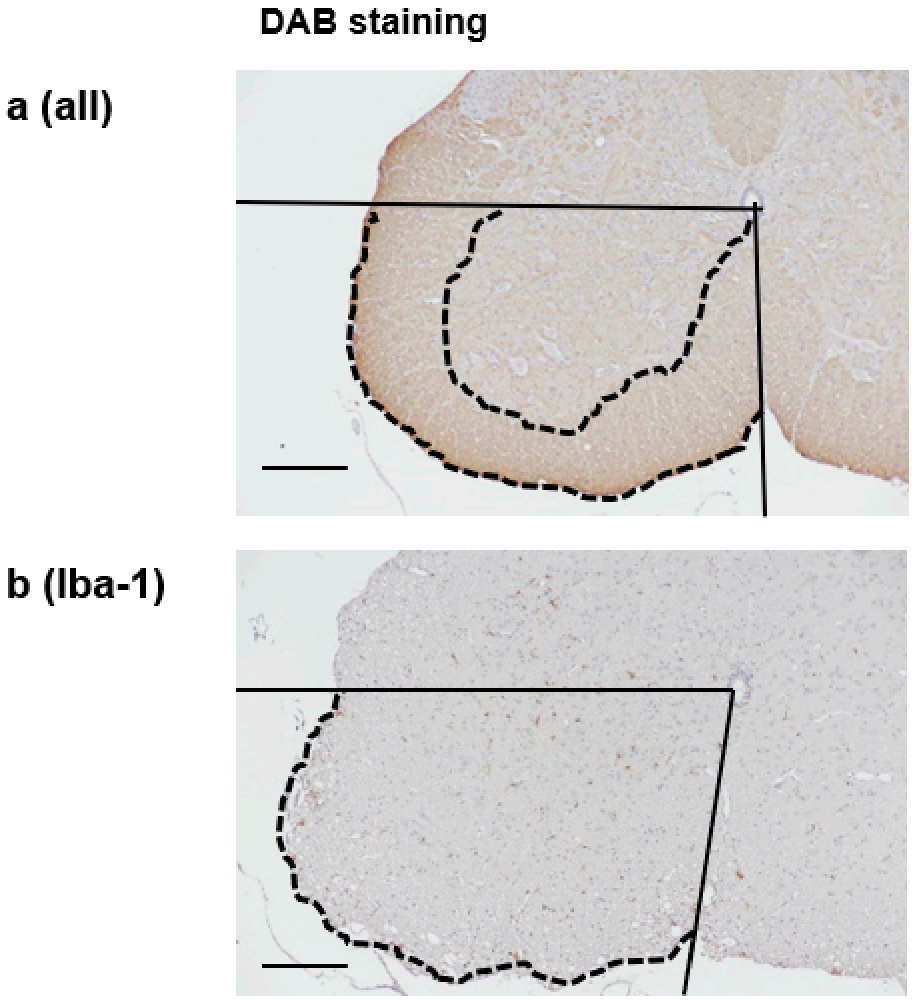

Figure S5. Example of quantification of immunohistochemically stained images. **(a)** Transverse spinal cord sections were divided by horizontal and vertical lines passing through the central canal (solid lines), and the central gray matter area was manually excluded to define the white matter region of interest (the area encircled by a dashed line). The positive-stained areas in this region were measured. This method was used for immunostaining analyses, except for Iba-1 staining. **(b)** Transverse spinal cord sections

were divided by horizontal and vertical lines passing through the central canal (solid lines), and the region of interest consisting of the central gray matter and white matter (the area encircled by a dashed line) was defined. The positive-stained areas in this region were measured. This method was used for Iba-1 staining. Scale bars: 100 µm.

Table S1. Antibodies used in this study

A common buffer was used for each antibody (5% normal goat serum + 1% bovine serum albumin in 50 mM Tris buffer).

**Antigen**	**Clone**	**Host**	**Dilution**	**Incubation temperature**	**Incubation time**	**Source**
MBP	Monoclonal	Mouse	1:200	4°C	Overnight	Santa Cruz
CD3	Polyclonal	Rabbit	1:500	4°C	Overnight	Abcam
F4/80	C1:A3-1	Rat	1:100	4°C	Overnight	Abcam
GFAP	G-A-5	Mouse IgG1	1:500	4°C	Overnight	Sigma
Iba-1	Polyclonal	Rabbit	1:500	4°C	Overnight	Wako

		IgG				
C3	11H9	Rat	1:50	4°C	Overnight	Hycult Biotech
S100A10	Monoclonal	Rabbit	1:50	4°C	Overnight	Abcam
iNOS	Monoclonal	Mouse	1:50	4°C	Overnight	Santa Cruz
Arg-1	43-81	Mouse	1:100	4°C	Overnight	Santa Cruz
IL-17A	Polyclonal	Rabbit	1:1000	4°C	Overnight	Abcam
